# Utilization of Cancer Cell Line Screening to Elucidate the Anticancer Activity and Biological Pathways Related to the Ruthenium-Based Therapeutic BOLD-100

**DOI:** 10.3390/cancers15010028

**Published:** 2022-12-21

**Authors:** Brian J. Park, Paromita Raha, Jim Pankovich, Mark Bazett

**Affiliations:** Bold Therapeutics Inc., 422 Richards St, Suite 170, Vancouver, BC V6B 2Z4, Canada

**Keywords:** BOLD-100, cell screening, ruthenium, cancer therapeutics, ribosomes

## Abstract

**Simple Summary:**

There is an unmet need for novel anticancer therapeutics that work differently to current standard-of-care therapies. BOLD-100 is a unique clinical-stage anticancer compound that is based on the rare metal, ruthenium. Understanding the bioactivity of BOLD-100 can accelerate its development towards approval and into clinical practice. The aim of this study was to use a large panel of cancer cell lines to formulate a sensitivity profile of BOLD-100 across various cancer types. BOLD-100 demonstrated increased activity in cell lines from esophageal cancer, blood cancers, and bladder cancer. These indications are in addition to the gastrointestinal cancers currently in clinical development, thus opening new opportunities. Using the sensitivity profile for downstream bioinformatics and pathway analysis revealed associations between cancer cell lines’ sensitivity to BOLD-100 and ribosomal gene expression, including several genes coding for large- and small-ribosomal subunits. These findings provide evidence that ribosomal processes may be a critical component of BOLD-100’s mechanism.

**Abstract:**

BOLD-100 (sodium trans-[tetrachlorobis(1H indazole)ruthenate(III)]) is a ruthenium-based anticancer compound currently in clinical development. The identification of cancer types that show increased sensitivity towards BOLD-100 can lead to improved developmental strategies. Sensitivity profiling can also identify mechanisms of action that are pertinent for the bioactivity of complex therapeutics. Sensitivity to BOLD-100 was measured in a 319-cancer-cell line panel spanning 24 tissues. BOLD-100’s sensitivity profile showed variation across the tissue lineages, including increased response in esophageal, bladder, and hematologic cancers. Multiple cancers, including esophageal, bile duct and colon cancer, had higher relative response to BOLD-100 than to cisplatin. Response to BOLD-100 showed only moderate correlation to anticancer compounds in the Genomics of Drug Sensitivity in Cancer (GDSC) database, as well as no clear theme in bioactivity of correlated hits, suggesting that BOLD-100 may have a differentiated therapeutic profile. The genomic modalities of cancer cell lines were modeled against the BOLD-100 sensitivity profile, which revealed that genes related to ribosomal processes were associated with sensitivity to BOLD-100. Machine learning modeling of the sensitivity profile to BOLD-100 and gene expression data provided moderative predictive value. These findings provide further mechanistic understanding around BOLD-100 and support its development for additional cancer types.

## 1. Introduction

Metal-based compounds have the potential to be highly efficacious anticancer therapies with novel mechanisms of action [1,2]. The properties of metal-based compounds, including the ability for ligand substitutions, a vast chemical structural space, complex geometries, multiple oxidation states, and potential metal-ligand interactions, provide extensive avenues for drug design and discovery [1,2,3,4]. The platinum-based therapeutic cisplatin, and its derivatives oxaliplatin and carboplatin, are mainstay chemotherapies extensively utilized in advanced cancer patient care [5,6]. However, intrinsic and acquired resistance to cisplatin and low response rates in advanced settings has triggered the search for novel metal-based alternatives with differentiated mechanisms and improved therapeutic outcomes [7]. The development of metal-based therapeutics has proceeded in a range of metals including gold, copper, iridium, and ruthenium [8,9,10,11]. Despite this, very few metal-based anticancer drugs have completed clinical development [12,13]. Therefore, advanced development strategies for metal-based compounds are needed to fulfill the therapeutic potential of this class of molecules.

Preclinical screening of novel therapeutics against panels of cancer cell lines remains a pertinent protocol in drug development [14]. Since the pioneering work of the NCI60 cell line panel, which sought to link drug sensitivity and cancer genotypes, large cancer cell line panels and subsequent pharmacogenomic analysis have become crucial in identifying development strategies and providing novel insights into mechanisms of action (MOA) [15,16]. The depth of knowledge and data around cancer cell lines have accelerated the advancements in accurate drug prediction tasks in oncology [17,18], with several consortia including the Genomics of Drug Sensitivity in Cancer (GDSC) project and the Cancer Therapeutics Response Portal (CTRP) providing screening data for over one thousand cancer cell lines in response to hundreds of chemical compounds [19,20]. Furthermore, the abundance of cell line molecular data available in public databases such as the Cancer Cell Line Encyclopedia (CCLE) allows for pharmacogenomic analyses to uncover molecular determinants of drug sensitivity [21]. Genomic modalities, including gene expression, protein expression, copy number variation, and somatic mutations, have been used in drug prediction tasks and can be utilized to understand potential drug targets and MOAs. Gene expression in particular is one of the most predictive modalities of drug response [22,23,24,25,26] and can provide insights into the drug MOA [27,28]. This facilitates the translation of in vitro findings in the laboratory to the clinic, which remains a rate-limiting step in novel metal-based drug development [29].

The ruthenium complex BOLD-100 is a clinical-stage anticancer compound that is composed of sodium trans-[tetrachlorobis(1H indazole) ruthenate(III)] with cesium as an intermediate salt form. Predecessor molecules include IT-139, NKP-1339, and KP1339. BOLD-100 has successfully completed a Phase 1 monotherapy trial and is currently in a Phase 1b/2 clinical trial in combination with chemotherapy in advanced gastrointestinal cancers (NCT04421820) [30]. Beyond gastrointestinal cancers, BOLD-100 has shown efficacy in a range of preclinical models, including breast, lung, and liver cancer [31,32,33]. Mechanistically, BOLD-100 has a complex, multifaceted MOA which includes the modulation of the endoplasmic reticulum (ER) chaperone 78 kDa glucose regulated protein (GRP78) in conjunction with ER stress, as well as the induction of reactive oxygen species (ROS) and a subsequent DNA damage response (DDR) [31,32,34,35,36,37]. Additionally, potential interactions between BOLD-100 and ribosomal proteins have been recognized, as well as the effect of BOLD-100 on altered cellular metabolism [38,39,40,41]. Collectively, both the optimal development strategy and the definitive MOA for BOLD-100 remains elusive.

The depth of knowledge and data yielded by in vitro cell line screening assays presents an opportunity to assess the efficacy of BOLD-100 across a large panel of cancer types. In this study, a cell viability screen across 319 cancer cell lines spanning 24 tissues of origin using BOLD-100 monotherapy was utilized to determine cancer types with increased sensitivity. In order to build the knowledge base of BOLD-100’s potential bioactivity, the sensitivity profile of BOLD-100 across the cancer cell lines was compared to anticancer compounds in the GDSC database. Utilizing the depth of cancer cell line genomic data from CCLE, a statistical modeling analysis was performed to identify candidate genes which associated with BOLD-100 susceptibility, identifying the importance of ribosomal gene expression for BOLD-100 response. Finally, a machine learning model was trained using the sensitivity profile of BOLD-100 and cell line genomic data in order to assess the ability to predict BOLD-100 sensitivity in cancer cell lines. This incorporation of cell line screening, pharmacogenomics, drug response modeling, and multivariate machine learning has formulated a more cohesive understanding of BOLD-100’s therapeutic potential and provides opportunities for future research for clinical applications.

## 2. Materials and Methods

### 2.1. Cancer Cell Screen

The effect of BOLD-100 (Bold Therapeutics Inc.; Vancouver, BC, Canada) and cisplatin (Qilu Pharmaceuticals; Jinan, China) on the growth inhibition in 319 cancer cell lines (Table S1) across 24 tissues of origin was evaluated (Crown Bioscience Inc.; Beijing, China). All cell lines were acquired and tested by Crown Bioscience Inc. Cell lines were seeded in 96-well plates and were subject to BOLD-100 or cisplatin treatment for 72 h in a Cell Titer-Glo Luminescent Cell Viability Assay (Promega; Madison, WI, USA). Initial viability measurements were obtained prior to drug treatment. For BOLD-100, a top concentration of 250–500 μM and two-fold serial dilutions in dimethyl sulfoxide (DMSO) were used to achieve nine dose levels. For cisplatin, a top concentration of 100 μM and 3.16-fold serial dilutions were used to achieve nine dose levels. Culture media was used as a means of vehicle control. Each treatment at each dose level per cell line was tested in triplicate. Cell culture media was based on recommended media for optimal growth of each cell line, with Dulbecco’s Modified Eagle Medium (DMEM) or Roswell Park Memorial Institute (RPMI1640) medium as the primary media used, supplemented with fetal bovine serum (FBS) (ExCell Bio; Shanghai, China). Envision Multi Label Reader 2104-0010A (Perkin Elmer; Waltham, MA, USA) was used for plate readout.

### 2.2. Calculation of BOLD-100 and Cisplatin Sensitivity Profile in Cancer Cell Lines

R package *PharmacoGx* (version 3.0.2) was used to fit a four-parameter log-logistic curve on the cell line viability data and compute the half maximal inhibitory concentration (IC_50_) [42]. The normalized measure of drug efficacy (GR_max_) was calculated using the online web tool *GRCalculator* (http://www.grcalculator.org/grcalculator/; accessed on 31 May 2021) [43]. Within-tissue median values for BOLD-100 and cisplatin IC_50_ and GR_max_ were calculated in order to compare the two drugs’ sensitivity profiles. The within-tissue distributions of IC_50_ and GR_max_ were visualized in boxplots, where the length of the boxes represent the interquartile range (IQR) (i.e., the range between 25th (Q1) and 75th (Q3) percentiles) and the bottom and top whiskers represent Q1 − 1.5xIQR and Q3 + 1.5xIQR, respectively, which denote the threshold for outliers. ANOVA was used to test whether BOLD-100 and cisplatin sensitivity profiles showed significant variability across the tissues of origin. Cell lines in the following tissues of origin were further classified into cancer subtypes according to data available in Cell Model Passports (https://cellmodelpassports.sanger.ac.uk/; accessed on 30 September 2021): blood, bone, brain, kidney, head and neck, thyroid, esophagus, lung, ovary, prostate, soft tissue, cervix, and uterus [44]. The following tissues were classified into subtypes according to the literature: bladder [45,46], breast [47], colon [48], and liver [49]. The BOLD-100 IC_50_ measurements for the following cell lines could not be obtained due to the values being out of the range of the concentrations tested: HT-1376 (bladder), SK-N-AS (brain and nerves), BT-483 (breast), HeLa, MS751 (cervix), and 22RV1 (prostate). Therefore, these cell lines were excluded in the analysis of BOLD-100 sensitivity. All statistics and data visualizations were performed using the R programming language (version 4.0.5). The centrality and dispersion of the drug sensitivity values were reported using the median and the IQR and comparison of IC_50_ distributions were carried out using non-parametric tests such as the Wilcoxon signed-rank test and the Kruskal-Wallis ANOVA.

### 2.3. Comparison of BOLD-100 and Cisplatin Sensitivity Profiles versus GDSC Database

Drug sensitivity profiles of anticancer compounds across cancer cell lines were obtained from the GDSC database (https://www.cancerrxgene.org/; accessed on 10 May 2021). Data from two separate drug screen cohorts termed *GDSC1* and *GDSC2* were collected. In cases where a drug entry appeared in both datasets, data from the *GDSC2* cohort was used, as per the database documentation [20,50]. This produced a dataset containing 449 drugs screened against 988 cancer cell lines. The initial dataset was then trimmed according to the following criteria: (1) cell lines not tested in the current BOLD-100/cisplatin cell panel were removed and (2) cell lines whose drug sensitivity data was missing in more than 50% of drugs tested were excluded. The result was a dataset of 412 drugs across 260 cancer cell lines. BOLD-100 and cisplatin IC_50_ data were log transformed and separated based on whether cell lines originated from solid or liquid tumors. Spearman rank correlation analysis was performed between BOLD-100 IC_50_ profile and the individual sensitivity profiles within the GDSC dataset in order to compare the BOLD-100 response signature to the 412 other drugs (Table S2). The Benjamini-Hochberg false discovery rate (FDR) correction was performed for multiple testing corrections. The statistical significance level α was set at 0.01. This analysis was repeated with the cisplatin IC_50_ profile. The associated pathway annotations for each drug were obtained from the GDSC database (accessed on 10 May 2021).

### 2.4. Identification of BOLD-100 Response Associated Genes

The sensitivity profile of BOLD-100 and cisplatin was tested for possible confounding variables in the experimental design. Variables included age, gender, doubling time, mutation rate, microsatellite instability (MSI) status, growth property (adherent, semi-adherent. Suspension), and cell culture media. The age, gender, doubling time, and mutation rate data for the cell lines were obtained from the CCLE database (https://sites.broadinstitute.org/ccle; accessed on 13 July 2021). The growth property and MSI status data were obtained from the study by Iorio et al. [50]. Using ordinary least squares (OLS) regression, the following were treated as numeric variables: age, mutation rate, and doubling time, while the following were encoded as categorical variables: gender, MSI status, growth property, and culture media. Cell lines were analyzed separately based on whether they were derived from liquid cancers or from solid cancers (Table S3).

For the modeling analysis, genomic data was obtained from the CCLE database (accessed on 31 May 2021), which included gene expression, mutations, metabolomics, and reverse-phase protein array data [27]. For the mutation data, 470 cancer genes were selected as per the list identified by Iorio et al. [50]. Each set of genomic data was modeled against BOLD-100 IC_50_ using a multiple regression model. The multiple regression model incorporated the following as the predictor variables for a given cell line: a given genomic feature *X*_1_, a categorical variable *X*_2_ representing the tissue of origin, and a categorical variable *X*_3_ representing the culture media used. The natural-log of the BOLD-100 IC_50_ was encoded as the continuous response variable *Y*. For gene expression, metabolomics, and protein expression data, the genomic features (*X*_1_) were treated as continuous variables. For mutation data, *X*_1_ were binarized based on the absence or the presence of a given mutation prior to regression. A generalized additive model method was used to fit the regression model using the R package *gam* (version 1.20.2). The Benjamini-Hochberg FDR correction was performed on the regression p-values (Table S4).

### 2.5. Functional Enrichment Analysis of BOLD-100 Related Genes

Significant gene expression associations (Benjamini-Hochberg FDR < 0.1) were used as input for functional enrichment analysis using the R package *clusterProfiler* (version 3.10.1) [51]. The Gene Ontology (GO) database was used for the pathway over-representation analysis, including the biological process (BP), molecular function (MF), and cellular component (CC) sub-ontologies (Table S5). A minimum pathway term size of 10 and a maximum size of 500 was used, with FDR cut-off of 0.05. Over-represented GO terms were summarized using the web tool *REVIGO* (http://revigo.irb.hr/; accessed on 31 May 2021) and the R package *treemap* (version 2.4.3) [52].

### 2.6. Development of Predictive Learning Model Using Gene Expression

To investigate whether cancer cell lines’ gene expression showed predictive ability in estimating BOLD-100 sensitivity, a nonlinear machine learning model was trained to predict BOLD-100 IC_50_. For this, a random forest regression model was used with the BOLD-100 IC_50_ obtained from the cell screen as the continuous target variable using the R package *caret* (version 6.0.92) [53]. For feature selection, the top 300 genes from the gene expression multiple regression model (sorted by FDR) were selected as predictors. An 80:20 training-testing split was used on the data. Hyperparameter tuning during model training was performed using a five-fold cross-validation on the training data prior to evaluation on the test data; this was performed to prevent overfitting and potential data information leak [54]. The fitted regression model was evaluated on the test data using R^2^ and root-mean-squared-error (RMSE). Using the Gini impurity method, genes with high influence on the model performance were identified. The model training and evaluation process was repeated using 500 and 600 features instead in order to investigate any potential biases of feature selection on model fitting and performance.

## 3. Results

### 3.1. BOLD-100 Response Shows Variability across Cancer Cell Lines’ Tissue of Origin

**Figure 1:**
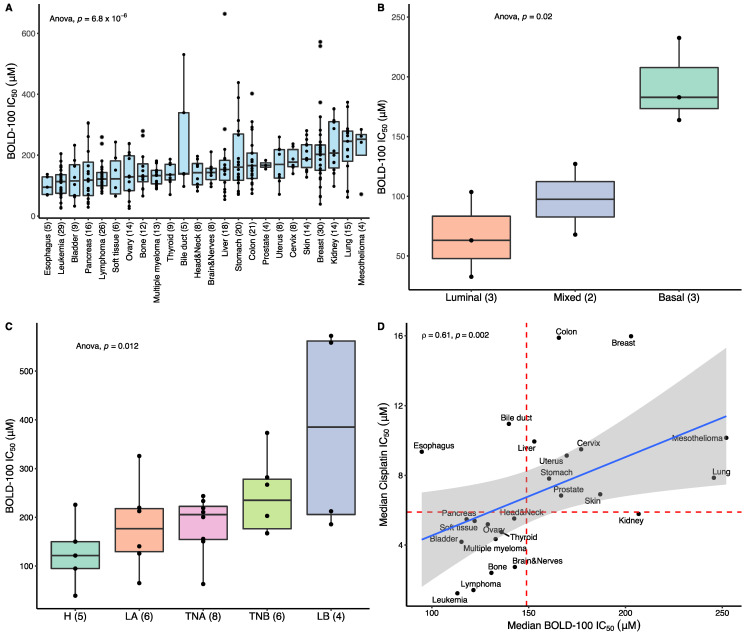
Sensitivity profile in BOLD-100 across a cancer cell line panel. 319 cancer cell lines were treated with BOLD-100 or cisplatin and the IC_50_ was calculated. (**A**) The IC_50_ distribution of BOLD-100 was grouped by tissue lineages and ranked by median IC_50_. X-axis label indicates the tissue lineage (number of cell lines). (**B**) The IC_50_ distribution of BOLD-100 across the subtypes of bladder cancer (*n* = 9), classified into basal, mixed, or luminal subtypes. The IC_50_ for one cell line (HT-1376) with a subtype classification of ‘mixed’ could not be computed as it was above the concentration range tested. (**C**) The IC_50_ distribution of BOLD-100 across the subtypes of breast cancer (*n* = 30), classified into luminal A (LA), luminal B (LB), HER2+ (H), triple negative A (TNA), and triple negative B (TNB). The IC_50_ for one cell line (BT-483) with a subtype classification of LA could not be computed as it was above the concentration range tested. (**D**) Comparison of the within-tissue IC_50_ median for BOLD-100 and cisplatin treatment. The red bisecting lines represent the pan-cancer median IC_50_ for each treatment across the cell panel (149 μM for BOLD-100 and 5.89 μM for cisplatin). The blue line represents the linear line of best fit between the two distributions. The gray shaded area represents the 95% confidence interval for the linear line of fit. Spearman correlation coefficient ρ and corresponding p-value is shown. For the box plots, the length of each box represents the range between the 25th and the 75th percentile whereas the whiskers represent the threshold for outlier values (**A**–**C**). For boxplots of cancer subtypes, the x-axis label indicates the subtypes (number of cell lines) (**B**,**C**).

To identify cancer types with greater susceptibility to BOLD-100, sensitivity profiles were obtained from 319 cancer cell lines and assessed based on tissues of origin. Non-cancerous cell lines were not assessed in this study. The distribution of BOLD-100 IC_50_ showed significant variability across the different tissues (ANOVA, *p* = 6.8 × 10^−6^) (Figure 1A), with values ranging from 25.1 μM to 664 μM and a median value of 149 μM (IQR = 98.4 μM), which is in line with previous reported values for this compound [31,37]. Esophagus, bladder, pancreas, and soft tissue cancer cell lines, as well as those from blood cancers showed lower IC_50_ with respect to the pan-cancer median. In contrast, lung, kidney, and breast cancer cell lines showed relatively higher median IC_50_. Cell lines derived from liquid cancers (i.e., leukemia, lymphoma, and multiple myeloma) showed a significantly lower median IC_50_ (120 μM, IQR = 52.4 μM) compared to cell lines derived from solid cancers (162 μM, IQR = 103 μM; Wilcoxon, *p* = 5.3 × 10^−7^).

Tissue-based classification does not encompass the complete complex nature of cancer [55]. Therefore, subclassifications within each tissue were tested for BOLD-100 sensitivity. Of the tissue types profiled in the cell panel, cell lines from bladder (*n* = 8; ANOVA, *p* = 0.02) and breast (*n* = 29; ANOVA, *p* = 0.012) showed a statistically significant variation in response to BOLD-100 across the defined subtypes (Figure 1B,C). In bladder cancer, the luminal subtype cell lines had the strongest response to BOLD-100, while HER2+ cell lines were the most responsive in breast cancer. These results suggest that certain subtypes within cancer tissues have a greater potential to benefit from BOLD-100 treatment.

Cancer cell line viability data in response to cisplatin was obtained and analyzed in parallel to BOLD-100. The distribution of cisplatin IC_50_ showed a statistically significant variability across the tissues of origin (ANOVA, *p* = 3.1 × 10^−6^) (Figure S1A). The range of IC_50_ was 0.177 μM to 105 μM, with a median of 5.89 μM (IQR = 9.21 μM), which was within the expected range for cisplatin [20,50]. Similarly to BOLD-100, liquid cancers showed relatively lower median IC_50_ in response to cisplatin (1.82 μM, IQR = 3.48 μM) compared to solid cancers (7.73 μM, IQR = 10.3 μM; Wilcoxon, *p* = 2.7 × 10^−15^). In within-tissue subtypes, the response to cisplatin showed a statistically significant variation within breast cancers (*n* = 30; ANOVA *p* = 0.0026) (Figure S1B), but, differing from BOLD-100, did not show a significant variability within the bladder cancer subtypes (*n* = 9) (Figure S1C). Cisplatin response did not show any significant within-tissue variability in other tissue types profiled (α < 0.05).

Within-tissue median IC_50_ values were used to directly compare the sensitivity profiles of BOLD-100 and cisplatin (Figure 1D). A moderately positive correlation was observed (ρ = 0.61, *p* = 0.002), suggesting that the majority of tissues shared similar susceptibility to BOLD-100 and cisplatin. However, esophagus, bile duct, liver, breast, and colon cancer cell lines showed a relatively greater degree of susceptibility to BOLD-100 compared to cisplatin. In breast cancer, the luminal A (LA) subtype showed the highest median IC_50_ in response to cisplatin (Figure S1C), while it showed the second lowest in response to BOLD-100. Therefore, even though the cell line panel showed largely concordant trends in relative responses to the two drug treatments, select cases which break this trend warrant further investigation and indicate potential leads for clinical development.

### 3.2. BOLD-100 Exhibits Differential Cytotoxic Effects across Cancer Indications

**Figure 2:**
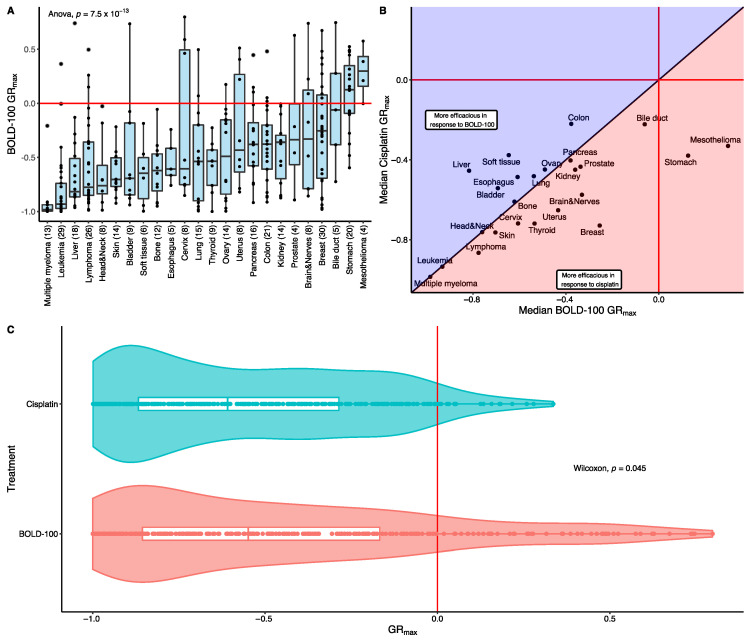
The cytotoxicity of BOLD-100 in the cancer cell line panel. (**A**) The GR_max_ distribution of BOLD-100 grouped by tissue lineages. The red horizontal line represents the threshold for cytotoxicity (i.e., GR_max_ = 0). The length of each box represents the range between the 25th and the 75th percentile whereas the whiskers represent the threshold for outlier values. The x-axis labels indicate the tissues of origin (number of cell lines). (**B**) The comparison of within-tissue median GR_max_ in response to BOLD-100 or cisplatin treatment. The bisecting diagonal line represents the decision boundary for which treatment exerts a more cytotoxic response. (**C**) The comparison of overall GR_max_ distribution across the entire cell panel in response to BOLD-100 or cisplatin. The vertical red line indicates the threshold for cytotoxicity (i.e., GR_max_ = 0).

Cytotoxicity involves the rapid killing of dividing cells, while cytostatic drugs impede cell growth via disruption of cell signaling and replication [56]. This distinction between the two types of therapy is not clear as many compounds display both effects in varying proportions [57]. However, the efficacy gain of cytotoxic therapy in cancer has been shown in both primary and metastatic tumors [58]. As both cytotoxicity and cytostaticity depend on the growth rate of dividing cells, representative metrics can be calculated to infer the degree of drug efficacy [43,59]. Evaluating both the potency (i.e., the IC_50_) and the efficacy (i.e., measure of cytotoxicity) thus provides additional dimensions in assessing the differential drug effect across disease types. Therefore, to infer the efficacy of BOLD-100, the growth rate metric GR_max_ was calculated using the cell screen viability data. GR_max_ is defined as the primary growth metric for drug efficacy and is bound by values −1 and +1, where positive values indicate partial growth inhibition, negative values indicate cytotoxic effects, and a value of 0 indicates a completely cytostatic effect [43,59]. Similarly to the IC_50_ values, the distribution of GR_max_ values in cell lines treated with BOLD-100 also showed statistically significant variability across the different tissues (ANOVA, *p* = 7.5 × 10^−13^) (Figure 2A). The majority of cell lines in the cell panel showed a degree of cytotoxicity in response to BOLD-100. Cells from blood cancers, as well as liver, bladder, and skin cancers tended to show higher degrees of cytotoxicity. Mesothelioma and stomach cancers were the only tissues that had positive median GR_max_ values, indicating BOLD-100 showed primarily cytostatic effects in these cell lines.

A comparison of the GR_max_ values between BOLD-100 and cisplatin showed that cell lines derived from liquid cancers tended to show the greatest degree of cytotoxicity in both treatments, which suggests that liquid cancers may be more predisposed to showing cytotoxic effects, or there may be assay biases related to cell growth conditions and drug exposure (Figure 2B). Cell lines derived from the liver, colon, soft tissues, bladder, and esophagus showed a larger extent of cytotoxicity in response to BOLD-100 than to cisplatin (Figure 2B). In contrast, tissues such as the breast, uterus, brain, and thyroid showed higher median GR_max_ values in response to cisplatin than to BOLD-100. This asymmetric behavior suggests the efficacy of the two treatments were not consistent with each other and such inconsistencies may favor the clinical use of BOLD-100 in select disease types. Furthermore, the overall distribution of GR_max_ across the entire cell panel in the two treatments showed a statistically significant difference (Wilcoxon, *p* = 0.045) with cisplatin showing a higher degree of cytotoxic activity (median GR_max_ = −0.609, IQR = 0.582) compared to BOLD-100 (median GR_max_ = −0.554, IQR = 0.677) (Figure 2C). Both distributions were skewed towards negative values with the BOLD-100 GR_max_ distribution exhibiting a longer tail at positive values. Despite the majority of cell lines showing a varying degree of cytotoxic activity in response to BOLD-100, the range of efficacy is broader compared to the cisplatin profile, suggesting that BOLD-100 has differential effects in certain cell lines.

### 3.3. BOLD-100 Response Shows Variability across Cell Culture Media

Variables related to the experimental design are known to affect sensitivity screening and can lead to misleading results and irreproducible findings [22,59,60,61]. Therefore, specific variables were tested for an association with the sensitivity to BOLD-100, including age, gender, microsatellite instability (MSI) status, doubling time, mutation rate, growth property (i.e., adherent, semi-adherent, or suspension), and culture media. OLS identified a significant association between BOLD-100 IC_50_ and culture media in both solid and liquid cancer derived cell lines (α < 0.05). In both stratifications, cells grown in RPMI + FBS media showed greater susceptibility to BOLD-100 based on the median IC_50_ (median of 139 μM in solid cancers and 112 μM in liquid cancers) compared to cells grown in DMEM+FBS media (median of 178 μM in solid cancers and 138 μM in liquid cancers). In contrast, comparing the IC_50_ of cisplatin across the two culture conditions showed no significant differences, which suggests this bias is related to BOLD-100 sensitivity. No other associations with BOLD-100 or cisplatin response were found across the confounding variables (Table S3).

### 3.4. BOLD-100 Response Profile Shows Weak Correlation to Other Known Drugs

A comparative analysis of drug sensitivity profiles allows for the identification of underlying similarities in the drugs’ MOA and has previously been used to infer the bioactivity of novel compounds [62,63,64]. To compare the sensitivity profile of BOLD-100 with other anticancer compounds, a correlation analysis was performed using the GDSC’s large-scale drug screening data. A total of 260 cancer cell lines with complete datasets were available for BOLD-100 cell screen and the GDSC dataset and were further stratified into solid or liquid cancers due to the previously observed increased response to BOLD-100 in liquid cancers. In solid cancer cell lines (*n* = 202), a total of 12 drugs’ sensitivity profiles showed a significant correlation with that of BOLD-100 (Table 1, Table S2 and Figure S2; Spearman rank correlation, α < 0.01). A parallel analysis in solid cancers with cisplatin identified 195 significantly correlated drug sensitivity profiles. In liquid cancer cell lines (*n* = 58), a total of 39 drugs’ sensitivity profiles showed significant correlations with BOLD-100 (Spearman rank correlation, α < 0.01), while 35 drugs were significantly correlated with cisplatin.

Cisplatin had a high degree of similarity to itself (solid tumors; ρ = 0.688; liquid tumors; ρ = 0.640) and the top associated drugs were exclusively from pathways related to DNA replication and genome integrity, which corresponds to the known mechanism of cisplatin’s cytotoxicity (Table 1) [7,65,66,67]. In solid cancer cell lines, BOLD-100 showed the largest degree of correlation to AT-7519, a cyclin-kinase inhibitor (ρ = 0.321) [68,69]. In liquid cancer derived cell lines, an inhibitor of the mutant KRAS-G12C protein showed the largest degree of correlation with BOLD-100 (ρ = 0.657). In both sets of analyses, there was no clear overarching theme in the correlated drugs to BOLD-100 with respect to their mode of action. This contrasting evidence suggests the MOA of BOLD-100 may be unique, multimodal, or complex in nature.

### 3.5. Multiple Regression Analysis Reveals Genes Associated to BOLD-100 Response

Multiple regression analyses were performed to identify potential associations between BOLD-100 sensitivity and various genomic modalities. For gene expression data analysis, a total of 294 cell lines were used. At a significance cut-off of α < 0.1, a total of 124 genes’ expression values showed an association with BOLD-100 response (Table S4). Using cancer driver mutations, metabolomics, and protein expression data from CCLE, multiple regression analysis did not return any significant associations with BOLD-100 sensitivity after FDR correction (α < 0.1). These results suggest that genomic modalities outside of gene expression showed no significant association with BOLD-100 sensitivity in the cell panel, at least in the scope of the data available in CCLE and the number of cell lines profiled in the current assay.

### 3.6. Pathway Enrichment Analysis of Associated Genes Reveals Key Biological Pathways

**Figure 3:**
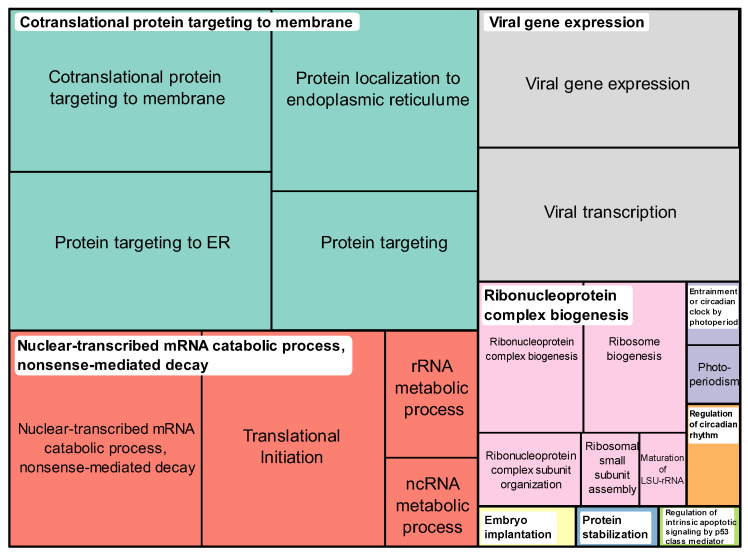
Over-represented Gene Ontology, biological process (GO:BP) terms in BOLD-100 sensitivity associated genes summarized by semantic similarity. Over-represented GO:BP terms (*n* = 38) were grouped and summarized via a treemap using *REVIGO*. The coloring of the individual tiles in the treemap represents family groupings based on term similarity while the size represents the size of the GO term in the database.

To identify underlying biological pathways related to genes associated with BOLD-100 sensitivity, the results from the gene expression multiple regression model (*n* = 124 genes) were used as input for functional enrichment analysis. Over-representation analysis using the GO annotation database returned a total of 51 enriched terms after FDR correction (α < 0.05) (Figure S3, Table S5). The top over-represented GO terms were associated with ribosomal processes, protein translation, and processes related to the ER. This over-representation was attributed to the abundance of genes coding for ribosomal proteins (RPs) including both large- and small-subunit proteins (RPLs and RPSs, respectively) present in the significant gene hit list (Table S4). To consolidate the pathway over-representation results, the GO analysis result was exported to the tool *REVIGO* for summarization based on representative pathways [52]. The *REVIGO* treemap based on the GO: BP over-representation result (*n* = 38) showed that the GO terms were grouped into the following families: processes related to protein targeting, RNA metabolic processes and translation, viral gene expression, and ribosomal biogenesis and assembly (Figure 3).

**Figure 4:**
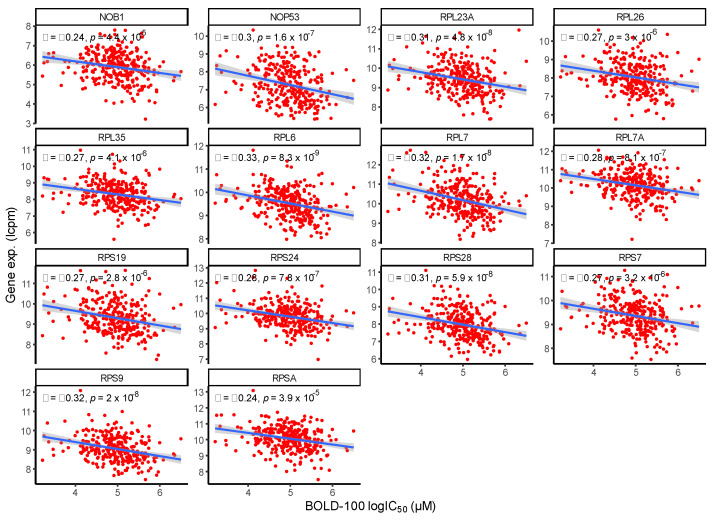
Correlation between genes related to ribosome biogenesis and BOLD-100 sensitivity in the cancer cell line panel. Significant genes within the Gene Ontology (GO) term GO:0042254—ribosome biogenesis showed consistent negative correlation with BOLD-100 IC_50_ from the cell panel. The x-axis denotes the natural log of BOLD-100 IC_50_ obtained from the cell panel. The y-axis denotes the normalized gene expression values for each gene in log-counts-per-million (lcpm), as per the data obtained from the Cancer Cell Line Encyclopedia (CCLE).

To elucidate the directionality of associations between BOLD-100 response and individual ribosomal gene expressions, correlation analyses were carried out (Figure 4). There was a consistently negative correlation between significant genes within the GO term GO:0042254: ribosome biogenesis and BOLD-100 IC_50_ from the cell panel. This finding suggests that the expression levels of genes associated with ribosome biogenesis are inversely related to the susceptibility to BOLD-100 treatment in the cell panel. Though the interaction between BOLD-100 and RPs have been elucidated in the past [38], the possibility of an association at the transcriptional level has not yet been studied; our current finding suggests there may be a predisposition in cell lines with higher levels of ribosome related genes to be more susceptible to BOLD-100 treatment.

### 3.7. Cell Line Gene Expression Data Shows Predictive Potential for BOLD-100 Response

**Figure 5:**
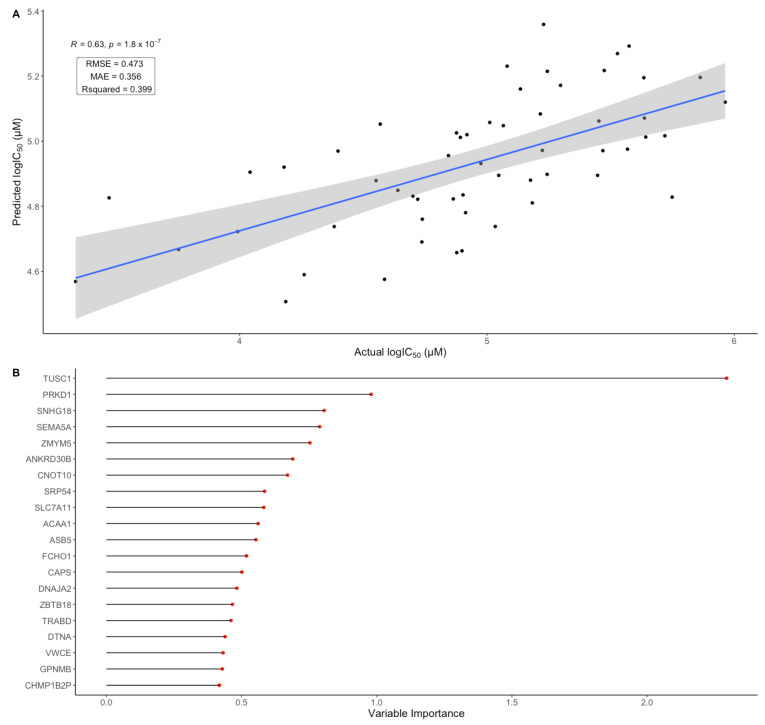
Evaluation of the random forest regression model fitting BOLD-100 sensitivity to gene expression data. (**A**) Model evaluation using the Pearson correlation between the model predicted BOLD-100 IC_50_ values on the test dataset and the measured IC_50_ values from the cell panel. Blue line represents the linear line of best fit and the gray shaded area represents the 95% confidence interval for the line of fit. The root-mean-squared-error (RMSE), mean-absolute-error (MAE), and R^2^ are reported for the model evaluation. (**B**) Variable importance of genes in the BOLD-100 IC_50_ prediction model. Top 20 features are shown. X-axis denotes the variable importance score, which is measured by the Gini impurity method.

Modeling techniques allow for drug response prediction using unseen data and the identification of variables with high predictive power [70,71]. In order to assess the potential of cancer cell line gene expression data in predicting BOLD-100 sensitivity, a machine learning model was trained to predict BOLD-100 IC_50_ in cancer cell lines. To select the number of features in the model (i.e., number of genes and their expression values), n = 300 genes sorted by statistical significance from the multiple regression model output were used for model training. Higher numbers of features (i.e., *n* = 500 and *n* = 600) were also tested and no significant differences in model performance was found (Figure S4). The model was trained on 80% of the cell line panel in response to BOLD-100 with their corresponding gene expression data. Hyperparameter tuning was performed using five-fold cross-validation within the training dataset; this reduced the possibility of overfitting and potential biases in the data partitions. The trained model was then tested on the remaining 20% of the cell panel data in order to obtain model evaluation metrics such as R^2^ and the RMSE by comparing the predicted IC_50_ values to the actual IC_50_ values measured from the viability assay. Using 300 features, the predictive model returned an R^2^ of 0.399 with an RMSE of log 0.473 μM (Figure 5A). This finding suggests that based on the BOLD-100 cell screen data, expression of select genes associated with BOLD-100 response had a moderate predictive potential in line with previous studies on drug response prediction models [22,23].

In a random forest model, variable importance describes a variable’s influence on output predictions. In ensemble tree models such as the random forest, this is typically measured by the drop in prediction accuracy after permutation of a given variable or the reduction in impurity in tree nodes that contain a given variable [72]. In order to identify pertinent genes in predicting BOLD-100 sensitivity, variable importance of each feature was extracted from the random forest model using the Gini impurity method. The tumor suppressor candidate 1 (TUSC1) gene scored the highest in terms of variable importance, which suggests its level of expression has the greatest influence on the model’s ability to predict BOLD-100 response (Figure 5B). Therefore, the prediction modeling work provides an additional dimension in the identification of genomic biomarkers that relate to BOLD-100 sensitivity.

## 4. Discussion

There is a significant unmet need for novel anticancer therapeutics, especially in advanced patients, that molecules such as BOLD-100 can address. In this study, a cell line screening assay of 319 cancer cell lines across 24 cancer types was completed in order to (1) identify possible cancer indications with greater susceptibility to BOLD-100 treatment and (2) generate a sensitivity profile of BOLD-100 to investigate its potential MOA. Specific cancer types, including esophageal, bladder and liquid cancers, were identified as having strong potential for further investigations. Using an integrated bioinformatics approach, this study also expanded the understanding of BOLD-100’s potential mechanism, with the identification of the importance of ribosomal proteins, thus providing avenues for future validation studies.

By screening broad categories of cancer types, this study supports the current clinical strategies with BOLD-100. BOLD-100 is currently being tested in a multiple-arm Phase 1b/2 clinical study (NCT04421820) in combination with fluorouracil, oxaliplatin and leucovorin (FOLFOX) for patients with advanced metastatic colorectal, bile duct, gastric, and pancreatic cancer. Encouragingly, this cell screen identified colorectal, bile duct, and pancreatic cancer as potential indications where BOLD-100 might have increased efficacy. Colorectal cancer has previously been identified as an indication with strong potential for BOLD-100 efficacy. In a previous Phase 1 monotherapy study of KP1339/IT-139 (a precursor version of BOLD-100 with the same active ingredient), the two patients with the largest decrease in target lesion size were both colorectal cancer patients [30]. This may be due to the differentiated resistance profile in colon cancer. In contract to BOLD-100, cisplatin had high intrinsic resistance in colon cancer cell lines in the cell screen, a phenotype which is reflected in the clinic [73]. Further, KP1019 (an imidazolium salt precursor to BOLD-100) does not have cross resistance to cisplatin in the A2780 ovary cisplatin resistant model [74]. Mechanistically, resistance to BOLD-100 has been linked to elevated glucose update and an increased lysosomal compartment [40], which differs from cisplatin’s primary resistance pathways that include transporters and DNA repair genes [75]. Collectively, the findings from the current study and early clinical findings both highlight BOLD-100’s differentiated clinical and resistance profile from cisplatin.

Cancer types with development potential were identified. Esophageal cancer is of particular interest due to it being the most responsive cancer type by median to BOLD-100 in the screen, while having high resistance to cisplatin. Within the esophageal cell lines, four out of five cell lines profiled were squamous cell carcinomas (SCCs), a cancer type that has low progression-free survival and overall survival for advanced patients, even with immunomodulating agents [76,77,78]. Liquid tumors were also identified as having an increased response to BOLD-100, reflecting earlier data on the precursor molecule KP1019 in an NCI60 screen [79]. Finally, bladder cancer is a potential development strategy, especially within the luminal subtype (Figure 1B). Although these new development directions are encouraging, cell screens can underrepresent certain cancer types, and do not fully recapitulate the clinical landscape, highlighting the need for additional validation work and investigations into additional cancer types [80,81,82,83]. Indeed, though the utilization of a cell panel may be limited in its ability to represent the complex landscape of cancer, current findings support the early clinical strategies in the development of BOLD-100 and provided potential new avenues for translational research.

The mechanism of action of BOLD-100 is likely to be multimodal and complex, with contributions from the regulation of multiple cellular stress pathways. Previous work on BOLD-100, and earlier generation molecules KP1339 (also referred to as NKP1339 and IT-139) and KP1019, have shown that ER stress and the downregulation of the stress regulator GRP78 are critical for BOLD-100’s efficacy [31,35,37]. In contrast, others have suggested ROS generation and DNA damage are the primary pathways involved in the anticancer effects of BOLD-100 [32,36]. Additional pathways including lipid metabolism have also been proposed [40,41]. Collectively, this suggests the presence of a multimodal mechanism that helps explain the results identified in this study. A considerably larger range of GR_max_ values with BOLD-100 compared to cisplatin was observed, suggesting that the pathways altered by BOLD-100 may be cell-line dependent. Similarly, comparison of the drug sensitivity profile against that of other compounds, a method that has previously been used to elucidate drug mechanisms [62,63], provided limited overlap to standard drugs. Differing from cisplatin, which was primarily associated with drugs related to DNA replication and genomic stability in concordance with its well-known mechanism of DNA chelation [5], BOLD-100’s association hits were across multiple drug classes, including cell cycle, DNA replication, and kinases. Of interest was a correlation of BOLD-100 to thapsigargin, a classical ER stress agent that has a very different impact on GRP78 expression but has similar downstream activation of the unfolded protein response (UPR) and C/EBP homologous protein (CHOP) induced apoptosis to BOLD-100 [31,84,85,86]. This association highlights the importance of ER stress as part of BOLD-100’s mechanism. The heterogeneity in correlated drugs with respect to their mode of action—as well as generally moderate correlation coefficients—suggests BOLD-100 may employ a complex, multimodal MOA dissimilar to a well-characterized compound such as cisplatin.

Ribosomal biogenesis was identified to be associated with the response to BOLD-100, with the expression levels of the RPs showing an inverse relationship with BOLD-100 sensitivity. In target identification studies, BOLD-100 was shown to bind RPLs and BOLD-100 increased expression of RPLs in treated HCT116 cells [38]. These results support the possible association between ribosomal processes and BOLD-100’s bioactivity. Indeed, the link between cytotoxic cancer therapy and the ribosome has been postulated in the past, with a variety of chemotherapeutic drugs targeting ribosome biogenesis [87,88]. Mechanistically, ribosomal stress is linked to downstream apoptosis via interactions between RPs and E3 ubiquitin-protein ligase (MDM2), which subsequently leads to P53 stabilization and cell cycle arrest [89]. This behavior has been shown to be dependent on protein kinase R (PKR)-like ER kinase (PERK), which is one of the downstream UPR effectors that BOLD-100 activates [31,90]. PERK induced phosphorylation of eukaryotic initiation factor 2ɑ (eIF2ɑ), also upregulated by BOLD-100, has been shown to cause the inhibition of ribosomal recycling and ribosomal disruption, which in turn enables RPs to sequester MDM2 from P53 [31,90]. The increasing evidence of an association between BOLD-100 and ribosomal processes suggests a potential role of the RP-MDM2-P53 axis in BOLD-100’s bioactivity. Ribosomal homeostasis and regulation are complex cellular processes and downstream validation analyses are required to identify the role of BOLD-100 in this context.

Drug sensitivity prediction modeling is an opportunity to develop unique response profiles for therapeutics. This study employed a simple generalized linear model as a means of feature selection and a classical machine learning model in the random forest regressor, which is a standardly utilized approach in the field of pharmacogenomics [23,91,92]. The model evaluation revealed an R^2^ of 0.399, which suggests the model explained the variability in the dataset to a reasonable degree, in line with the previous literature on drug sensitivity prediction tasks using random forests [92]. In pharmacogenomic models such as the one deployed in this study, collinearity between predictor variables (i.e., correlation between individual gene expression levels) may introduce biases in variable importance calculations [93]. However, there is evidence in the literature that genetic markers associated with interactions with other markers can be more efficiently identified using the random forest model [94]. Indeed, the advantage of the random forest compared to a univariate model is that variable importance captures the impact of not only the predictors on their own but also of interactions with other predictor variables. The top identified variable TUSC1 is a putative tumor suppressor gene [95] with no previously identified relationship to BOLD-100 response, and follow-up experiments are needed to elucidate the impact of this relationship. The results suggest that the in vitro BOLD-100 response profile can be modeled by readily available gene expression data with minimal feature engineering or preprocessing.

The cell screening approach and subsequent bioinformatics analysis using gene expression provided a wealth of new information around BOLD-100. However, no significant associations in the other genomic datasets were identified, including oncogene or tumor suppressor gene mutations or protein expression patterns. Gene expression data has been shown to be the most predictive modality in drug sensitivity prediction tasks [22,23,24]. Further, chemotherapeutics such as BOLD-100 are less likely to have significant association than targeted therapies in cell screens [50]. Additionally, Iorio et al. illustrated that larger cell screens are more likely to identify significant associations. They showed that a reduction from 1001 cell lines to 500 cell lines caused an ~80% loss in the number of statistically significant associations, a number that still exceeds the size of this study’s cell panel [50]. Although we cannot exclude the potential of unidentified molecular markers in hypersensitive cells driving the results, the response profile of BOLD-100 aligns with the current clinical strategies in bile duct, colorectal, and pancreatic cancer indications. This provides an encouraging outlook in the translation of BOLD-100 efficacy to patient populations. Collectively, the efforts outlined in this study have elucidated some of the complex features of BOLD-100’s mode of action, which provides hypotheses for further validation studies in more clinically relevant models.

## 5. Conclusions

The current study employed a cancer cell line panel to infer the differential sensitivity of BOLD-100 across different tissues and build a sensitivity profile that can be used for downstream pathway analysis. This enabled the finding of potential routes for clinical development of BOLD-100 in cancer types which were not identified previously, including esophageal, bladder, and liquid cancers. BOLD-100 generally exhibited cytotoxic effects across the cell panel and shared moderate correlations to a heterogeneous group of anticancer drugs found in the literature, with no clear overarching theme in bioactivity. Furthermore, potential molecular determinants of BOLD-100 sensitivity were identified, namely the association with genes coding for RPs and related pathways. This study thus provides a platform for translational research in BOLD-100 to expand the current findings to in vivo models and additional avenues towards next-generation machine learning tasks in oncology and drug prediction.

## Figures and Tables

**Table 1:** Spearman correlation results between sensitivity profiles of anticancer compounds from the Genomics of Drug Sensitivity in Cancer (GDSC) database and BOLD-100 and cisplatin profile from the cell panel. Associated pathway annotated for each drug was obtained from the GDSC database.

BOLD-100 vs. GDSC—Solid Cancers			
Drug	Pathway Name	ρ	FDR
AT-7519	Cell cycle	0.321	0.00290
Bleomycin	DNA replication	0.316	0.00290
Thapsigargin	Other	0.314	0.00290
FMK	Other, kinases	0.310	0.00327
Bosutinib	Other, kinases	0.291	0.00327
**BOLD-100 vs. GDSC—Liquid Cancers**			
**Drug**	**Pathway Name**	**ρ**	**FDR**
KRAS (G12C) Inhibitor-12	ERK MAPK signaling	0.658	0.000698
ULK1_4989	Other, kinases	0.640	0.000698
VSP34_8731	Other	0.635	0.000698
Vincristine	Mitosis	0.626	0.000698
Carmustine	DNA replication	0.622	0.000698
**Cisplatin vs. GDSC—Solid Cancers**			
**Drug**	**Pathway Name**	**ρ**	**FDR**
Cisplatin	DNA replication	0.688	1.54 × 10^−24^
Camptothecin	DNA replication	0.547	6.68 × 10^−14^
Mitoxantrone	DNA replication	0.516	7.45 × 10^−11^
Topotecan	DNA replication	0.509	1.21 × 10^−10^
Irinotecan	DNA replication	0.473	5.94 × 10^−10^
**Cisplatin vs GDSC—Liquid Cancers**			
**Drug**	**Pathway Name**	**ρ**	**FDR**
Epirubicin	DNA replication	0.792	1.41 × 10^−8^
Talazoparib	Genome integrity	0.776	1.48 × 10^−9^
PARP_9482	Genome integrity	0.756	1.41 × 10^−8^
PARP_0108	Genome integrity	0.747	1.46 × 10^−8^
Camptothecin	DNA replication	0.729	3.74 × 10^−7^

## Data Availability

All original contributions presented in the study, including generated data and analysis outputs are included in the article/supplementary material. Publicly available datasets were analyzed from the GDSC (https://www.cancerrxgene.org/) and CCLE (https://sites.broadinstitute.org/ccle).

## Supplementary Materials

The following supporting information can be downloaded at: https://www.mdpi.com/article/10.3390/cancers15010028/s1, Figure S1: The sensitivity profile of cisplatin across the 319 cancer cell line panel. Figure S2: Correlation between BOLD-100/cisplatin sensitivity profile from the cell panel and drugs from the Genomics of Drug Sensitivity in Cancer (GDSC) database. Figure S3: Over-represented Gene Ontology (GO) terms in BOLD-100 sensitivity associated genes. Figure S4: Performance of the random forest regression model trained on Cancer Cell Line Encyclopedia (CCLE) gene expression data and BOLD-100 IC_50_ from the cell panel. Table S1: Sensitivity profile of BOLD-100 and cisplatin across the cancer cell line panel. Table S2: Correlation analysis results between BOLD-100/Cisplatin sensitivity profile to the drugs from Genomics of Drug Sensitivity in Cancer (GDSC) database. Table S3: Linear regression result between possible confounding variables and BOLD-100 sensitivity profile across cell line panel. Table S4: Multiple regression results between BOLD-100 sensitivity profile and various genomic modalities from Cancer Cell Line Encyclopedia (CCLE). Table S5: Gene Ontology over-representation analysis result using genes (*n* = 124) associated with BOLD-100 sensitivity.
