# Supplementary material for: Utilization of Cancer Cell Line Screening to Elucidate the Anticancer Activity and Biological Pathways Related to the Ruthenium-Based Therapeutic BOLD-100
Source: Cancers (Basel). 2022 Dec 21;15(1):28. doi: 10.3390/cancers15010028 (PMC9817855; doi:10.3390/cancers15010028)
Supplement: Supplementary file 1 [file cancers-15-00028-s001.zip › Supplementary Figures.pdf]

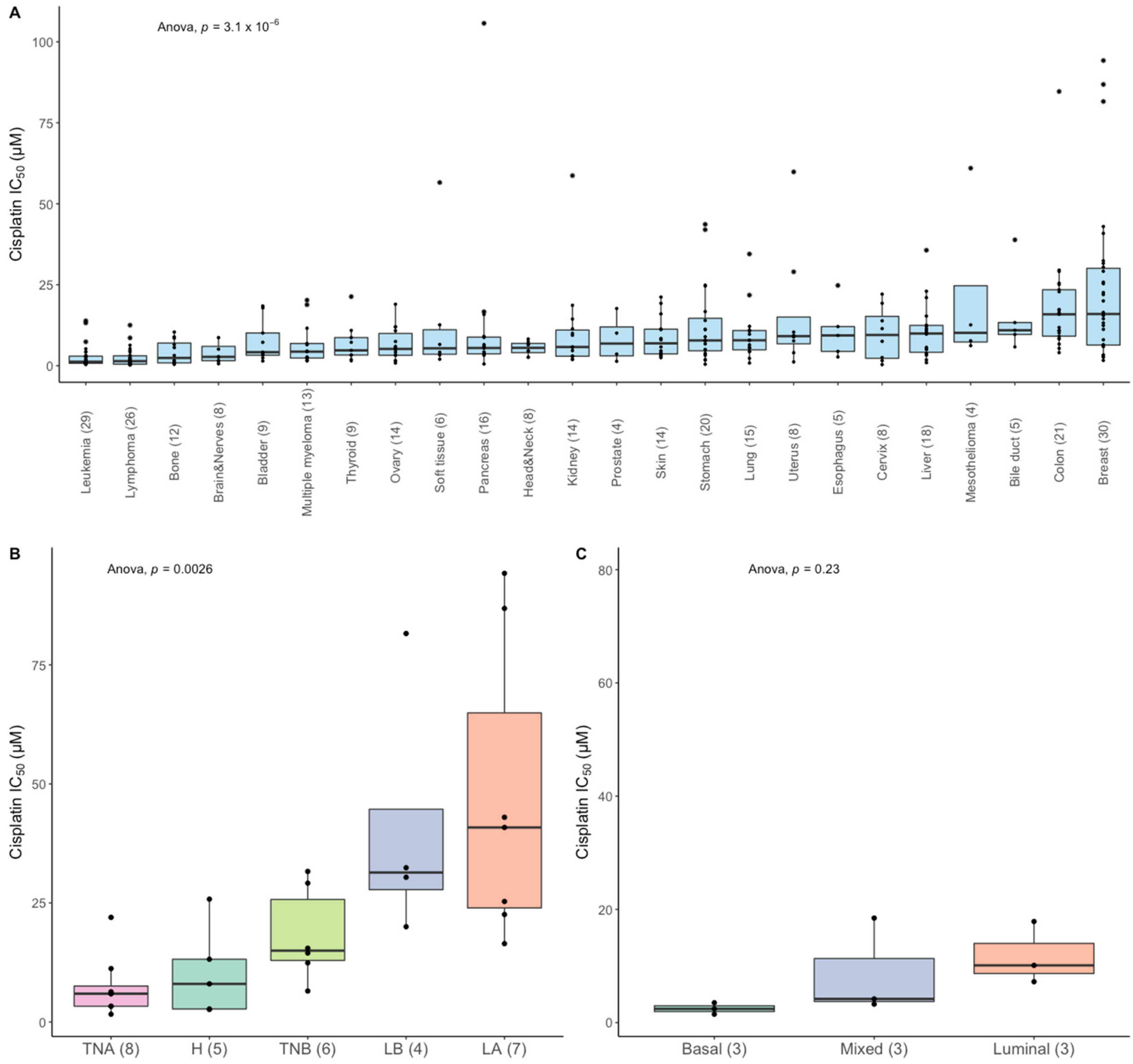

Figure S1. The sensitivity profile of cisplatin across the 319 cancer cell line panel. 319 cancer cell lines were treated with cisplatin and the viability readout was used to calculate the IC_{50}. A) The IC_{50} distribution of cisplatin across 24 tissues of origin. B) The IC_{50} distribution of cisplatin across the cancer subtypes of bladder cancer: basal, luminal, and mixed. C) The IC_{50} distribution of cisplatin across the cancer subtypes of breast cancer: luminal A (LA), luminal B (LB), $HER2^{+}$, triple negative A (TNA), and triple negative B (TNB). For the boxplots, the length of each box represents the distance between the 25th and the 75th percentile in the data distribution while the whiskers represent the thresholds for outliers. The x-axis labels represent the tissue lineages (number of cell lines) (**A**) or the cancer subtypes (number of cell lines) (**B, C**).

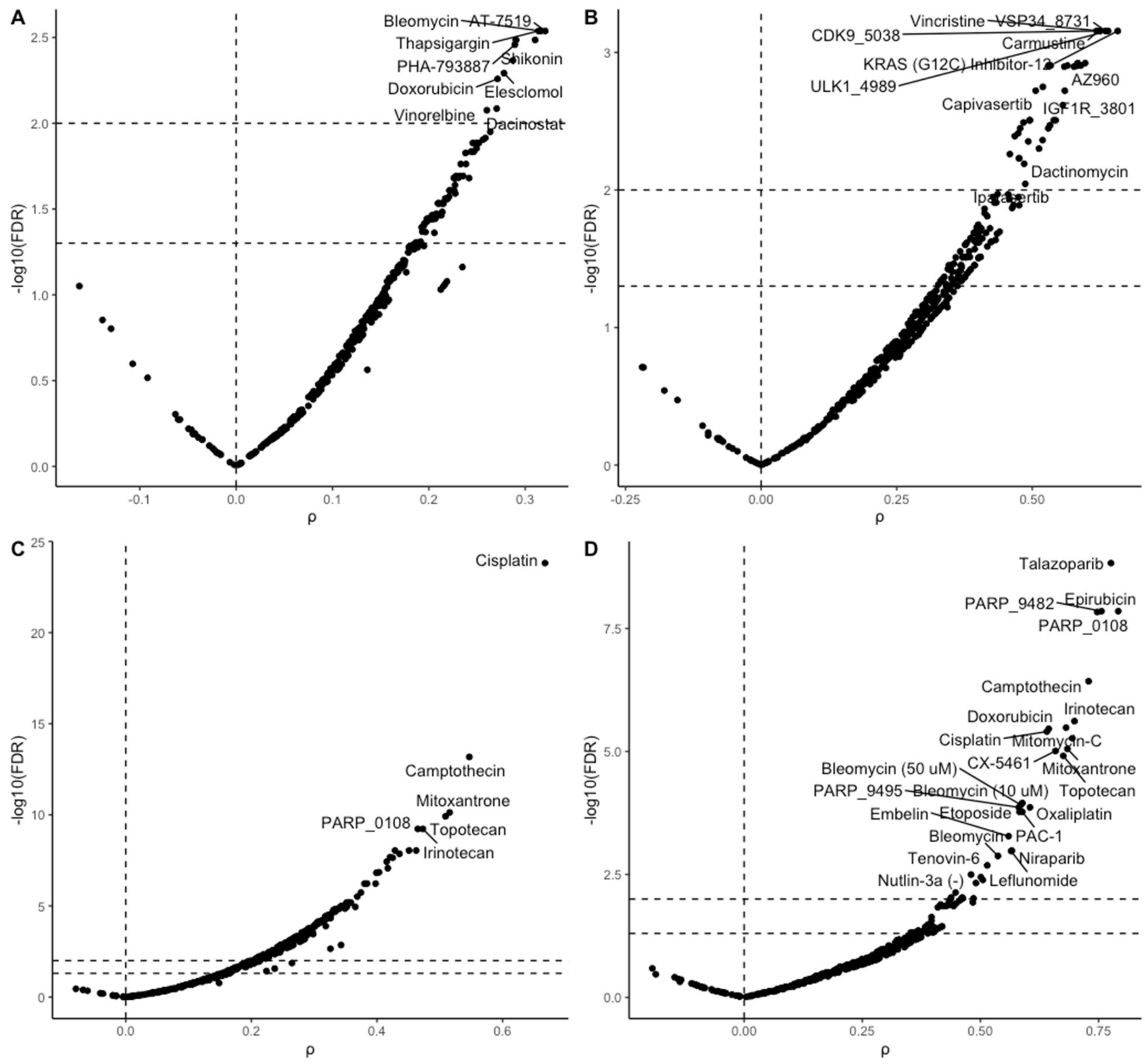

Figure S2. Correlation between BOLD-100/cisplatin sensitivity profile from the cell panel and drugs from the Genomics of Drug Sensitivity in Cancer (GDSC) database. The IC_{50} profiles obtained from the cell line panel were compared with known compounds from the GDSC drug screening data. Spearman correlation coefficients are shown with respect to statistical significance in analysis between GDSC data and A) BOLD-100 sensitivity profile in solid cancer cells, B) BOLD-100 sensitivity profile in liquid cancer cells, C) cisplatin sensitivity profile in solid cancer cells, and D) cisplatin sensitivity profile in liquid cancer cells. .

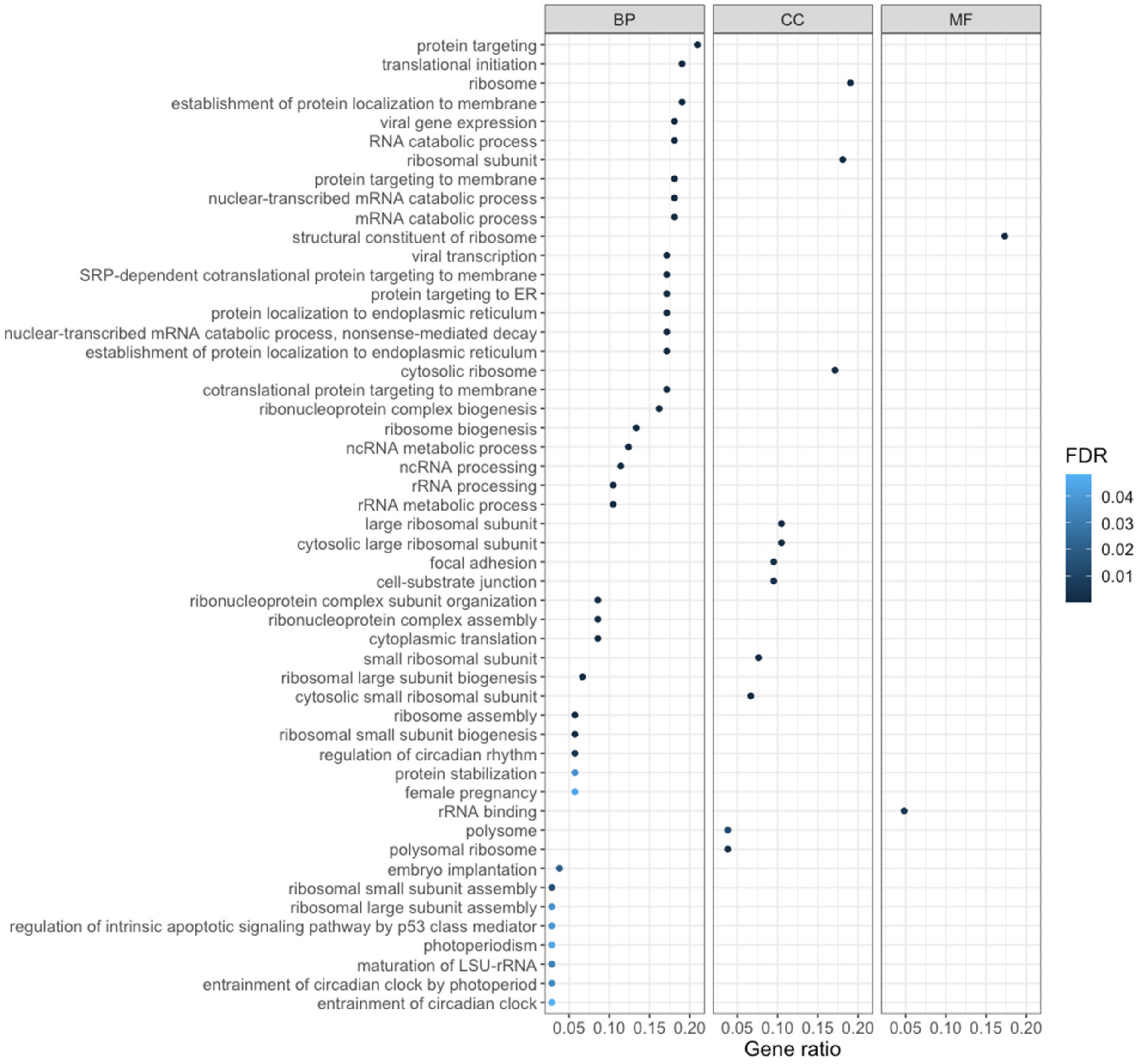

Figure S3. Over-represented Gene Ontology (GO) terms in BOLD-100 sensitivity associated genes. A total of 51 terms were found over-represented using the GO annotation database (false discovery rate (FDR) < 0.05). Individual terms are ranked by their gene ratio, defined as the number of intersecting genes from the input gene list with the particular gene set. Results are shown across the three sub-ontologies: biological process (BP; $n = 38$), cellular component (CC; $n = 11$), and molecular function (MF; $n = 2$).

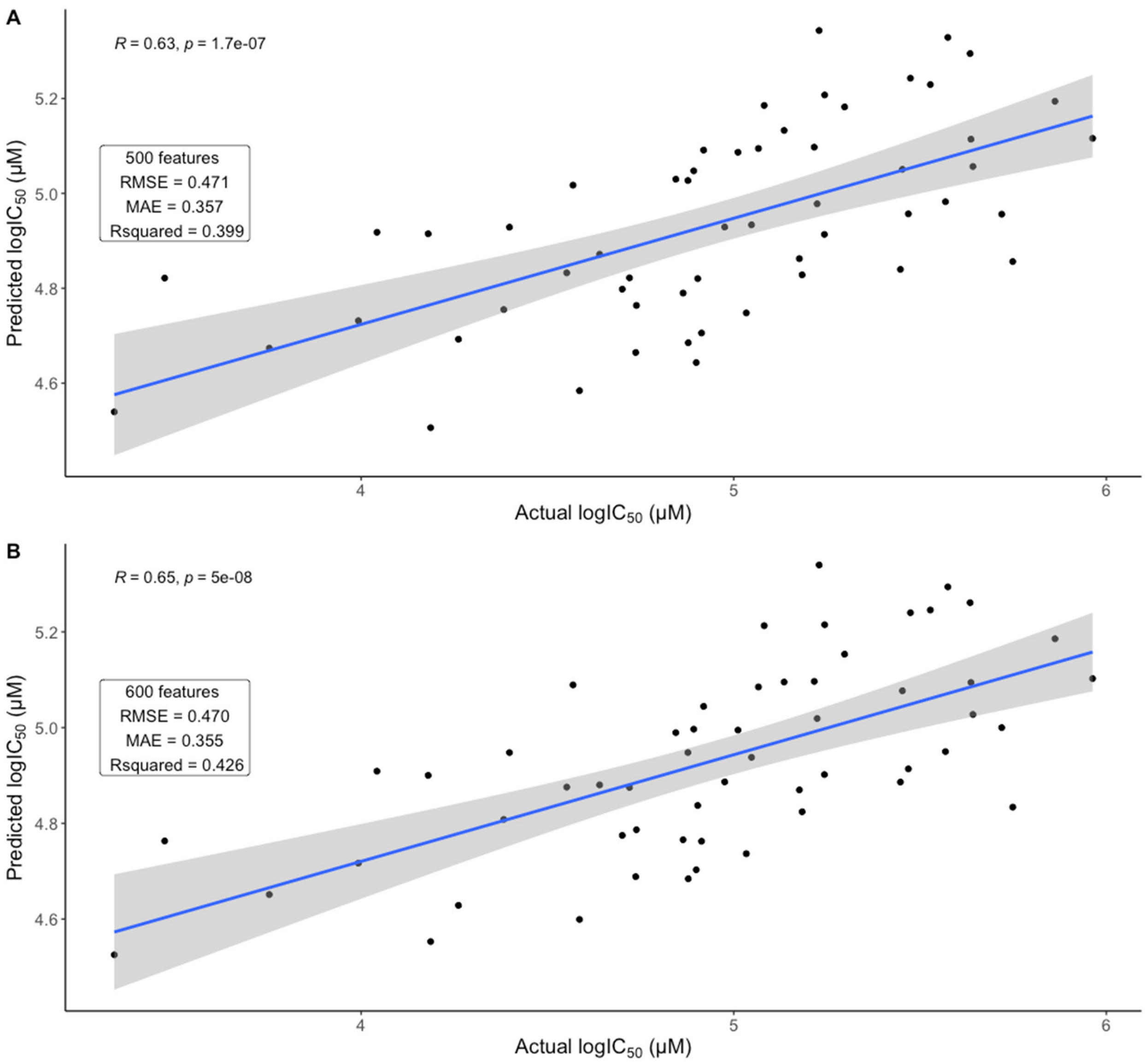

Figure S4. Performance of the random forest regression model trained on Cancer Cell Line Encyclopedia (CCLE) gene expression data and BOLD-100 IC_{50} from the cell panel. A) Evaluation of the regression model using the Pearson correlation between model predicted IC_{50} values versus actual; IC_{50} values from the BOLD-100 cell panel. The model was trained on 500 top genes sorted by statistical significance in relation to BOLD-100 sensitivity. B) Evaluation of the regression model using 600 top genes instead.
